# Is the 77.1 % rate of in-hospital mortality in patients receiving venoarterial extracorporeal membrane oxygenation really that high?

**DOI:** 10.1186/s13054-016-1379-1

**Published:** 2016-07-15

**Authors:** Kentaro Shimizu, Hiroshi Ogura

**Affiliations:** Department of Traumatology and Acute Critical Medicine, Osaka University Graduate School of Medicine, 2-15 Yamadaoka, Suita City, Osaka 565-0871 Japan

Aso and colleagues concluded that a nationwide study showed ‘high mortality rates’ in patients who received venoarterial extracorporeal membrane oxygenation (VA-ECMO), particularly in those with cardiogenic shock and cardiac arrest [1]. They stated that in-hospital mortality in these patients was 77.1 %. We believe that some comparisons should be made to determine whether the mortality rate is really that high.

ECMO, especially percutaneous cardiopulmonary support (PCPS), has been used extensively in emergency medicine and surgery as a simple but powerful device since 1987 in Japan [2]. Emergency cardiopulmonary support has been used in a variety of emergency cases and improved resuscitation. Maekawa et al. reported that, in a propensity-matched study of 162 out-of-hospital cardiac arrest patients, neurologic outcome in the extracorporeal cardiopulmonary resuscitation (CPR) group was improved compared with that in the conventional CPR group. In this study, CPR duration averaged 49 min, the ICU survival rate was 95.8 %, and the cerebral performance category status 1 or 2 at 3 months was 29.2 % [3]. This device has a beneficial effect on out-of-hospital cardiac arrest patients.

In the in-hospital cardiac arrest setting, Chen et al. reported that, in a propensity analysis of 113 cardiac arrest patients, extracorporeal CPR resulted in a higher survival rate to discharge than conventional CPR (28.8 % versus 12.3 %) [4]. Shin et al. reported that, in a propensity score matching analysis of 90 patients with cardiogenic cardiac arrest, in-hospital survival was higher with extracorporeal CPR (35.5 % versus 8.8 %) [5]. These reports suggested that in-hospital cardiac arrest ‘survival rates’ are around 10–20 % even in the hospital where extracorporeal CPR could be used. In the Aso et al. report [1], the ‘mortality’ of cardiac arrest patients in cardiogenic shock was 77.1 %. This percentage also included out-of-hospital cardiac arrests, so the in-hospital cardiac arrest mortality could be lower. Furthermore, physicians mostly use ECMO as a last-ditch measure because this procedure may have lethal adverse vascular effects and is very expensive. Even in such a critical situation, about one quarter of the patients survived to discharge in this nationwide database. From the viewpoint of physicians, this mortality rate does not appear high. These results could suggest that VA-ECMO may be a potential therapy for in-hospital cardiac arrest patients.
