# Supplementary material for: Is the 77.1 % rate of in-hospital mortality in patients receiving venoarterial extracorporeal membrane oxygenation really that high?
Source: Crit Care. 2016 Jul 15;20:202. doi: 10.1186/s13054-016-1379-1 (PMC4946148; doi:10.1186/s13054-016-1379-1)
Supplement: Related texts

# Reply: Authors’ response

**Authors:** Shotaro Aso, Hideo Yasunaga

We thank Dr. Shimizu and Dr. Ogura for their insightful comments. As they pointed out, the eligible patients in our study included out-of-hospital and in-hospital cardiac arrest patients, and the reported mortality rate (77.1 %) may be higher than that for in-hospital cardiac arrest patients alone. We agree that physicians mostly use VA-ECMO as a last-ditch measure. The aim of the present study was to clarify the current status of cardiac arrest patients treated with VA-ECMO using a national inpatient database. A previous review reported that the in-hospital mortality rate of cardiogenic shock patients with cardiac arrest varied widely, ranging from 6 % to 59 %, because of differences in population selection [6]. The previous studies cited by Dr. Shimizu and Dr. Ogura are limited because of their small sample sizes from single centers [3–5]. Our data were based on a national database that covers approximately 92 % of all tertiary-care emergency hospitals in Japan. Therefore, our results are more reliable than those in the previous studies.

We agree with Dr. Shimizu and Dr. Ogura that VA-ECMO may be a potential therapy for in-hospital cardiac arrest patients. However, the present study did not provide any evidence on the effectiveness of VA-ECMO because of the lack of a control group.

Dr. Shimizu and Dr. Ogura commented that “from the viewpoint of physicians, this mortality rate does not appear high.” However, we do not believe that this rate is low. The discrepancy may simply depend on differences in our senses of values. We believe that the important point is to acknowledge that there is much room for improvement in this rate, and again we would like to emphasize that further studies are needed to investigate the effects of VA-ECMO.

## Abbreviations

CPR, cardiopulmonary resuscitation; ECPR, extracorporeal cardiopulmonary resuscitation; PCPS, percutaneous cardiopulmonary support; VA-ECMO, venoarterial extracorporeal membrane oxygenation
